# Phosphates

**Published:** 1873-06

**Authors:** W. H. Jackson

**Affiliations:** Ann Arbor, Mich.


### PHOSPHATES.

BY W. H. JACKSON, ANN ARBOR, MICH.

Miss ——— presented March, 1872, age 16, bilious temperament, growing fast, not in very good health, desired to have her teeth filled. Upon examination found the molars badly decayed The enamel on the labial and buccal surfaces of all the teeth, for half the distance from the gums to the cutting and grinding surfaces, was so soft that it could be scraped with the finger nail, also the same action on the lingual and palatal surfaces of the molars and bicuspids, but to a less extent; color, white and chalky. Mucus gave decided acid reaction, as tested by blue litmus. I told her, if the secretions could not be changed, it would be of no use to fill her teeth. The molars were in such a condition that it would not answer to leave them. I declined to commence them, unless she would submit to a course of treatment, and follow it up a long time. She promised to do as I wished.

I then filled five common and one pulp cavity. I prescribed phosphate of lime, gr. ij, to be taken with each morning meal.

At the expiration of a month she presented herself a second time. I found that the disease was going no further. I continued the former prescription; told her to brush her teeth with a soft brush, and just before going to bed to rub a little cretæ precip. around the teeth, and leave it there. Did not see her again until November. Asked her if she had continued taking the phosphate as directed. She said she continued it about six weeks. After I saw her again, when her family physician (Homœopathic) heard of it, and told her she "must not take any more of it, for it would get into her bones," she was somewhat alarmed, and of course stopped taking it, but neglected to inform me. Nevertheless her health as well as her appetite improved, and continued to do so up to the time she called. Found the enamel of the incisors, cuspids, and bicuspids, hard where it had been affected, with a slight dark tinge, as if they had not been properly cleaned, with transverse furrows across them. The molars still showed a diseased condition, but not near as marked as at first.

At the recommendation of Dr. H. White, he having used the same with good results, I now prescribed Winchester's preparation of Churchill's Hypophosphites of Lime and Soda ; dose, one teaspoonful with each morning meal.

The patient called again in March, 1873. Found teeth much improved ; continued same prescription, it being more agreeable to take, not noticeable when taken in a glass of cold water. Called May 5th, no trace of disease left, except the slight dark tinge and furrows, which show where the disease had been.

Were this the only case I should not take the liberty to place it before you. A single case is not sufficient evidence of the efficacy of any remedy, neither should we recommend a remedy until we first test its value as a remedial agent in a multiplicity of cases.

I have reported this case as one of many, and I hope other practitioners will try this remedy, as I have met with many happy results from its use during the past four years ; not that phosphate of lime is a specific for all vitiated secretions, but that many times teeth can be preserved by its use that would otherwise be lost without it.

